# Novel Solutions to the Three-Anchor ToA-Based Three-Dimensional Positioning Problem

**DOI:** 10.3390/s21217325

**Published:** 2021-11-03

**Authors:** Mohamed Khalaf-Allah

**Affiliations:** Institute of Traffic Telematics, Technische Universität Dresden, 01062 Dresden, Germany; mohamed.khalaf_allah@tu-dresden.de; Tel.: +49-351-463-36786

**Keywords:** time of arrival (ToA), three-dimensional (3D) positioning, direct method (DM), particle filter (PF), unmanned aerial vehicle (UAV)

## Abstract

At least four non-coplanar anchor nodes (ANs) are required for the time-of-arrival (ToA)-based three-dimensional (3D) positioning to enable unique position estimation. Direct method (DM) and particle filter (PF) algorithms were developed to address the three-anchor ToA-based 3D positioning problem. The proposed DM reduces this problem to the solution of a quadratic equation, exploiting the knowledge about the workspace, to first estimate the *x*- or *z*-coordinate, and then the remaining two coordinates. The implemented PF uses 1000 particles to represent the posterior probability density function (PDF) of the AN’s 3D position. The prediction step generates new particles by a resampling procedure. The ToA measurements determine the importance of these particles to enable updating the posterior PDF and estimating the 3D position of the AN. Simulation results corroborate the viability of the developed DM and PF algorithms, in terms of accuracy and computational cost, in the pursuit and circumnavigation scenarios, and even with a horizontally coplanar arrangement of the three ANs. Therefore, it is possible to enable applications requiring real-time positioning, such as unmanned aerial vehicle (UAV) autonomous docking and circling a stationary (or moving) position, without the need for an excessive number of ANs.

## 1. Introduction

Position determination is a fundamental issue in many fields [1] such as wireless sensor networks [2,3], mobile communications [4,5,6], telecommunications [7], multiple-input multiple-output (MIMO) radar [8], sonar [9] as well as human–computer interaction [10]. Time of arrival (ToA), time difference of arrival (TDoA), time sum of arrival (TSoA), received signal strength (RSS), and angle of arrival (AoA) are commonly used measurements with anchor nodes (ANs) at known locations for positioning a user tag or target. The distance information can be obtained from ToA, TDoA, TSoA, and RSS measurements, while AoA measurements provide bearing information. In the two-dimensional (2D)/three-dimensional (3D) spaces, position determination using ToA or RSS, TDoA, TSoA, and AoA measurements refer to determining the intersection of circles/spheres, hyperbolas/hyperboloids, ellipses/ellipsoids, and straight lines, respectively [1]. The positioning problem is a nontrivial task since the user tag position is nonlinear in these measurements.

The typical time-based measurements used for positioning are ToA, TDoA, and TSoA. The distance information is obtained by multiplying these measurements by the known signal propagation speed in the medium. TSoA is similar to TDoA but is not particularly common [11]. TSoA arises in MIMO [8] and multistatic [12] systems consisting of two sets of ANs (or sensors), i.e., transmitters and receivers [1]. Radiofrequency (RF)-based positioning systems usually use wireless technologies such as Wi-Fi [13,14,15,16], ZigBee [17,18], Bluetooth [19,20,21], ultra-wideband (UWB) [22,23,24], radiofrequency identification (RFID) [25,26,27,28], pseudolites [29], the fifth-generation (5G) communication system [30], and millimeter-wave (mmWave) and terahertz (THz) frequency bands in the envisioned sixth-generation (6G) communication networks [31]. Further technologies used for positioning include visible light communication (VLC) [32], ultrasound [33,34], acoustic [35,36], infrared [37,38], vision [39], magnetic field [40], and dead reckoning [41]. With the rise of 5G networks and beyond, the internet of things (IoT), and unmanned aerial vehicles (UAVs), the research on positioning approaches remains an active field and continues to receive more attention. Three-dimensional position estimation is one of the unique features desired in next-generation applications, while most published algorithms focus on 2D position estimation. The main focus of this article is the ToA-based 3D positioning.

The ToA-based positioning approach requires a synchronized system in which transmitters, e.g., ANs, and the receiver, e.g., user tag, have a common clock, or a system in which all units are transceivers that can estimate the round-trip time (RTT), also known as the round time of flight (RToF), [42,43] to avoid the synchronization issue. In RToF systems, i.e., two-way ranging (TWR) [44], one unit (say user tag) sends a signal to a second unit (say AN), which immediately transmits the signal back. Thus, the RToF is proportional to twice the propagation time in addition to the processing time required at the AN. The synchronized system using UWB signals delivers very accurate ToA estimates [45]. However, achieving synchronization between all units is generally difficult and expensive. The alternative transceiver system is less expensive and simpler to implement, but delivers, in general, less accurate ToA estimates. In the self-positioning scenario, signal processing and position estimation are accomplished at the user tag. In the opposite scenario, i.e., remote positioning or tracking, the ANs perform signal processing and position estimation. The mathematical procedure for position estimation can be equally applied to both scenarios. The positioning algorithms apply to various systems irrespective of the type of signal being radio, acoustic, or optical.

The diverse civilian applications of UAVs include [46,47,48] communication relaying, traffic monitoring, firefighting, security, plant protection, logistics, reconnaissance, maritime observation, wildlife monitoring, search and rescue, remote sensing, photography, filmmaking, demining, emergency response [49], precision agriculture [50], mapping [51], civil engineering [52], law enforcement [53], and scientific research [54]. UAVs have increasing benefits in supply chain logistics, inventory applications [55,56,57,58], distribution centers [59,60,61], and in metropolitan areas [62] due to their capacity to ship up to six-kilogram payloads, up to sixteen-kilometer ranges [63]. Therefore, UAV-based package delivery is one of the most featured applications [64,65]. The Federal Aviation Administration (FAA) has finalized the operational rules for routine commercial use of small unmanned aircraft systems (UAS) [66]. It is anticipated that the number of UAVs will surpass conventional aircraft traffic by 2035 [67]. Accurate positioning and navigation of UAVs are key requirements in most applications [58,68].

Quadrotors have many advantages in the emerging field of UAV applications due to their maneuverability, motion control, fixed-point hovering, and vertical take-off and landing (VTOL) capability. Autonomous (waypoint) navigation, hovering, and autonomous landing of quadrotors in unstructured indoor environments is still a major issue that must be resolved by providing a reliable positioning solution to enable complex maneuvers and trajectory tracking. It is necessary to design and implement multiple functions to realize any UAV application [69]. A UAV-based light show application requires several functions, e.g., path planning to determine the movements according to the choreographic design, positioning, flight control, and power management to move the UAVs according to the planned path and for take-off and safe landing [69]. Furthermore, management and coordination may also require networking and communications functions.

The accurate positioning of UAVs is the objective of the positioning function. For outdoor applications, the dominating scheme is the use of a global navigation satellite system (GNSS), e.g., the global positioning system (GPS). Low-cost GNSS receivers provide meter-level accuracies, which is not sufficient for, e.g., land surveying and elegant outdoor light shows. To enhance positioning and reach centimeter-level accuracies outdoors, UAVs should be equipped with real-time kinematic (RTK) capability. Autonomous docking of UAVs consists of two phases: autonomous approach and autonomous landing. Vision-based techniques are quite common for autonomous docking [70]. However, due to the limited field of view (FoV), other methods should be integrated, especially in GNSS-denied environments. In [71], UWB-based positioning is used during the approaching phase. The landing pad is detected by the onboard vision system and the landing maneuver is accomplished by integrating the UWB and vision measurements.

Although position determination is a well-studied problem, there is still room to improve the state-of-the-art. The development of improvements aims at either reducing the computational costs while keeping the positioning accuracy as high as possible, increasing the positioning accuracy while keeping the computational costs as low as possible, or reducing the hardware costs of the positioning system while minimally impacting the accuracy, as well as developing practical numerical solutions to intractable problems from the mathematical point of view.

In this article, the 3D position determination based on minimal, i.e., three, ToA measurements is addressed. Two-dimensional/three-dimensional ToA-based positioning requires at least three non-collinear/four non-coplanar ANs, respectively, to guarantee a unique solution [11,72,73]. One less AN, i.e., two/three ANs for the 2D/3D cases, respectively, can be sufficient to have a unique solution in a few certain scenarios, e.g., if the user tag is equidistant from two/three non-coplanar ANs in the 2D/3D cases, respectively. Thus, the positioning of a user tag in an *N*-dimensional (*N*-D) space using *N* ToA measurements from *N* ANs is still an open problem [74]. To the best knowledge of the author, there is no solution available in the open literature to uniquely solve the 3D positioning problem with three ToA measurements from three ANs. To improve the state-of-the-art, we propose a direct method (DM) and a particle filter (PF) real-time solutions for the three-anchor ToA-based 3D positioning problem without the need for an initial position guess.

Quasi-horizontal coplanar arrangements of ANs provide poor vertical position observability [75,76,77,78,79]. Therefore, the vertical position of the user tag cannot be estimated accurately and, thus, 3D positioning would be infeasible. This is in line with the well-known wisdom that accurate positioning is obtained when the user tag is within the convex hull of the ANs [1,80,81]. It will be demonstrated that 3D positioning with three (quasi-)horizontally coplanar ANs, i.e., poor vertical position observability, can be accomplished using the developed DM, i.e., exact solution, and PF, i.e., by searching the correct state space, to enable resolution of the vertical position ambiguity.

Thus, the contribution of the article is two-fold: (1) To demonstrate the successful application of the developed DM and PF to solve the three-anchor ToA-based 3D positioning problem; (2) To demonstrate the capability of the developed DM and PF to solve the same problem even when the three ANs are horizontally (quasi-)coplanar.

Any non-colinear arrangement of three ANs defines a plane. In this article, coplanarity (or quasi-coplanarity) is restricted only to a horizontal plane for brevity. Thus, three (quasi-)coplanar ANs refer to a horizontal plane defined by their arrangement, and three non-coplanar ANs indicate that the plane defined by their arrangement is not horizontal.

The remainder of the article is organized as follows: Section 2 reviews related algorithms on ToA-based positioning. The DM algorithm and the PF solution to the three-anchor ToA-based 3D positioning problem are developed in Section 3 and Section 4, respectively. Simulation results are presented and discussed in Section 5, and the study is concluded in Section 6.

## 2. ToA-Based Positioning Algorithms

ToA-based position estimation is usually accomplished by optimizing, i.e., maximizing or minimizing, an objective function. Common objective functions include posterior density functions, likelihood functions, the sum of squared measurement residuals, risk functions, and robust loss functions [82]. Least-squares (LS) and maximum likelihood (ML) are the most commonly used estimation criteria. The LS estimation does not require any knowledge about the measurement probability density function (PDF) unlike the ML estimation, which requires either exhaustive global search using grid search or random search approaches such as genetic algorithms [83] and particle swarm optimization [84], or computationally intensive local search using iterative algorithms. If measurement errors are assumed to be zero-mean white Gaussian with known variances, the inverse of these variances constitutes the elements of a diagonal weighting matrix used to obtain the weighted LS (WLS) estimate, which is equivalent to the ML estimate and attains the Cramer–Rao lower bound (CRLB) [85]. In the 3D case, the CRLB exists when at least four non-coplanar ANs are used [86].

Iterative LS algorithms solve the ToA-based positioning problem by iterative descent techniques such as the steepest-descent method, the Newton method, the Gauss-Newton method, the Levenberg-Marquardt method, and the trust-region method [82]. These iterative descent techniques are linear inference methods, i.e., the estimate of the position is updated after each iteration, starting from the initial guess, based on the rule defined by the specific technique. The final position estimation is accomplished upon convergence. These algorithms generally deliver similar estimates, provided they converge successfully [82]. Iterative algorithms require an initial guess of the unknown parameter (user tag position) to start. The initial guess must be chosen as close to the true user tag position as possible to guarantee convergence, with minimal iterations, to the true solution especially if multiple local optima are existing. In indoor environments, it is not always possible to find a suitable initial guess that works for all scenarios [82]. An initial guess can be usually obtained by the solution of a closed-form (CF) algorithm or a DM [87]. In certain good tag-anchors geometries, the geometrical center of the ANs can be used as the initial guess. Multiple local minima may exist in nonlinear LS 3D positioning problems. Therefore, (close to) coplanar ANs geometries should be avoided [82] to enable convergence to the global minimum, i.e., the true user tag position. The computational cost of the iterative algorithms can be an issue for low-cost and low-power applications.

In general, strict non-iterative LS solutions to nonlinear problems, e.g., the ToA-based positioning problem, do not exist. However, closed-form (CF), i.e., non-iterative, LS positioning solutions can be developed with some simplifications or assumptions, e.g., linearization techniques, which ease the computational requirements at the expense of accuracy [82]. Many CF LS positioning algorithms are documented in the open literature across many fields, e.g., signal processing, wireless communication, radar, aerospace, etc. Many developed algorithms are identical, though their derivations are often different [82]. CF algorithms provide computationally efficient solutions with suboptimal accuracies due to the introduced simplifications, thus, are attractive for low-power and low-cost applications. Many CF LS solutions were developed for ToA and RSS systems, i.e., range or distance measurements, e.g., [88,89,90,91,92,93], and for TDoA systems, i.e., range-difference measurements, e.g., [94,95,96,97,98,99,100,101,102,103,104,105,106]. All these algorithms perform squaring on the range and range-difference equations to remove the square root. Linear and nonlinear terms of the position coordinates are obtained due to squaring. The nonlinear term is also treated as an unknown parameter, called the nuisance parameter, and is estimated along with the position coordinates of the user tag. Therefore, ToA-based CF LS algorithms require at least four non-coplanar ANs for 3D positioning. The accuracy of position estimation can be enhanced by exploiting the relation between the estimated position and the nuisance parameters [107,108,109].

Another important class of algorithms is the DM [87], which are algebraic, i.e., exact, solutions to the ToA-based positioning problem. The accuracy of DMs depends heavily on the accuracy of the measurements. DMs work with a certain number of measurements, usually the minimum number of measurements required to solve the positioning problem, and, thus, do not make use of any additional measurements when available. The ToA-based DM developed in [87] requires exactly four non-coplanar ANs to estimate the 3D position of the user tag. In [110], the use of three distance measurement equipment (DME) stations was investigated for estimating the absolute altitude of airplanes by a DM algorithm. An accuracy of about 9 m was achieved at a measurement update rate of 10 Hz. The DMs do not involve any matrix operations, do not need an initial guess of the user tag position to run, and do not require any assumptions about the distribution of the measurement errors. Furthermore, DMs are the basis of data compression techniques, since they reduce computational, storage, and communication requirements, and allow simple fusion with other sensors. Therefore, DMs are suitable for applications with limited computational and power resources and can be used when knowledge about the characteristics of measurement errors cannot be obtained. Moreover, DMs can provide a good initial guess to start/initialize iterative positioning algorithms or to restart a stranded PF.

## 3. Direct Method (DM) for ToA-Based Three-Dimensional (3D) Positioning

Radio signals, which are electromagnetic waves, propagate at the speed of light and arrive at the receiving antennas with some time delay, proportional to the range, i.e., distance, between the transmitter and receiver antennas. The time delay, τ, is defined τ=d/c, where d is the distance between the transmitter and receiver antennas and c is the speed of light. The estimates of τ are ToA measurements. Multiplying ToA measurements by c generates distance measurements.

Consider 3D positioning of a user tag or target using three ToA measurements from three ANs, and assume that the ToA measurements are realized by a synchronization mechanism or by a TWR scheme. In a 3D Cartesian coordinate system, the known 3D position of AN *i*, i∈{1,2, 3}, is defined $a_{i}={\left[x_{i},y_{i},z_{i}\right]}^{T}\in {\mathbb{R}}^{3}$, and the unknown user tag 3D position, at time $t_{k}$, is defined $x_{k}={\left[x_{k},y_{k},z_{k}\right]}^{T}\in {\mathbb{R}}^{3}$. Assuming that at any time instant, $t_{k}$, ToA measurements from the three ANs are available, the *i*-th distance measurement, $d_{k,i}$, i∈{1,2, 3}, is, thus, defined

(1) $$d_{k,i}=∥a_{i}-x_{k}∥+n_{k,i},\;i\in \left\{1,2,\;3\right\},$$

where ∥·∥ denotes the Euclidean vector norm and $n_{k,i}\sim N\left(0,\sigma_{k,i}^{2}\right)$ is the zero-mean Gaussian distance measurement noise with variance $\sigma_{k,i}^{2}$. The zero-mean Gaussian noise is an adequate assumption after proper calibration of the positioning system. The true range or distance, $r_{k,i}=∥a_{i}-x_{k}∥$, from the user tag to AN *i*, i∈{1,2, 3}, at time instant, $t_{k}$, is written

(2) $$r_{k,i}=\sqrt{{\left(x_{k}-x_{i}\right)}^{2}+{\left(y_{k}-y_{i}\right)}^{2}+{\left(z_{k}-z_{i}\right)}^{2}},\;i\in \left\{1,2,\;3\right\}.$$

Squaring Equation (2) and substituting the subscript *i*, we obtain the set of three equations

(3) $$r_{k,1}^{2}={\left(x_{k}-x_{1}\right)}^{2}+{\left(y_{k}-y_{1}\right)}^{2}+{\left(z_{k}-z_{1}\right)}^{2},$$

(4) $$r_{k,2}^{2}={\left(x_{k}-x_{2}\right)}^{2}+{\left(y_{k}-y_{2}\right)}^{2}+{\left(z_{k}-z_{2}\right)}^{2},$$

(5) $$r_{k,3}^{2}={\left(x_{k}-x_{3}\right)}^{2}+{\left(y_{k}-y_{3}\right)}^{2}+{\left(z_{k}-z_{3}\right)}^{2}.$$

DM solutions with three non-coplanar and three coplanar ANs are developed in Section 3.1 and Section 3.2, respectively.

### 3.1. DM Solution with Three Non-Coplanar Anchor Nodes (ANs)

Subtract Equations (4) and (5) from Equation (3) and let $x_{21}=x_{2}-x_{1}$, $x_{31}=x_{3}-x_{1}$, $y_{21}=y_{2}-y_{1}$, $y_{31}=y_{3}-y_{1}$, $z_{21}=z_{2}-z_{1}$, and $z_{31}=z_{3}-z_{1}$, to obtain the following two equations

(6) $$x_{21}\cdot x_{k}+y_{21}\cdot y_{k}+z_{21}\cdot z_{k}=\frac{1}{2}A_{1},$$

(7) $$x_{31}\cdot x_{k}+y_{31}\cdot y_{k}+z_{31}\cdot z_{k}=\frac{1}{2}A_{2},$$

where $A_{1}=\left(r_{k,1}^{2}-r_{k,2}^{2}\right)+\left(x_{2}^{2}-x_{1}^{2}\right)+\left(y_{2}^{2}-y_{1}^{2}\right)+\left(z_{2}^{2}-z_{1}^{2}\right)$ and $A_{2}=\left(r_{k,1}^{2}-r_{k,3}^{2}\right)+\left(x_{3}^{2}-x_{1}^{2}\right)+\left(y_{3}^{2}-y_{1}^{2}\right)+\left(z_{3}^{2}-z_{1}^{2}\right)$. Solving Equations (6) and (7) for $z_{k}$, we obtain

(8) $$z_{k}=\frac{1}{z_{21}}\left(\frac{1}{2}A_{1}-x_{21}\cdot x_{k}-y_{21}\cdot y_{k}\right),$$

(9) $$z_{k}=\frac{1}{z_{31}}\left(\frac{1}{2}A_{2}-x_{31}\cdot x_{k}-y_{31}\cdot y_{k}\right).$$

The right-hand side (RHS) of Equations (8) and (9) are equal. Thus, $z_{k}$ can be canceled out to obtain an explicit expression for $y_{k}$ in terms of $x_{k}$ as

(10) $$y_{k}=\alpha_{1}+\beta_{1}\cdot x_{k},$$

where $\alpha_{1}=\frac{1}{2}\left(z_{21}\cdot A_{2}-z_{31}\cdot A_{1}\right)/\left(y_{31}\cdot z_{21}-y_{21}\cdot z_{31}\right)$ and $\beta_{1}=\left(x_{21}\cdot z_{31}-x_{31}\cdot z_{21}\right)/\left(y_{31}\cdot z_{21}-y_{21}\cdot z_{31}\right)$.

Similarly, $y_{k}$ can be canceled out by solving Equations (6) and (7) for $y_{k}$ to obtain an explicit expression for $z_{k}$ in terms of $x_{k}$ as

(11) $$z_{k}=\alpha_{2}+\beta_{2}\cdot x_{k},$$

where $\alpha_{2}=\frac{1}{2}\left(y_{21}\cdot A_{2}-y_{31}\cdot A_{1}\right)/\left(y_{21}\cdot z_{31}-y_{31}\cdot z_{21}\right)$ and $\beta_{2}=\left(x_{21}\cdot y_{31}-x_{31}\cdot y_{21}\right)/\left(y_{21}\cdot z_{31}-y_{31}\cdot z_{21}\right)$.

Substituting Equations (10) and (11) in Equation (3), we obtain two solutions for $x_{k}$ as

(12) $$x_{k}=-\frac{b_{1}\pm \sqrt{b_{1}^{2}-4a_{1}c_{1}}}{2a_{1}},$$

where $a_{1}=\beta_{1}^{2}+\beta_{2}^{2}+1$, $b_{1}=2\beta_{1}\left(\alpha_{1}-y_{1}\right)+2\beta_{2}\left(\alpha_{2}-z_{1}\right)-2x_{1}$, and $c_{1}={\left(\alpha_{1}-y_{1}\right)}^{2}+{\left(\alpha_{2}-z_{1}\right)}^{2}+x_{1}^{2}-r_{k,1}^{2}$. The ambiguity in Equation (12) can be resolved by exploiting available information about $x_{k}$, e.g., a solution is rejected if it is placed outside the workspace. After accepting a solution for $x_{k}$ from Equation (12), the DM 3D solution is completed by solving Equations (10) and (11).

The existence of dual solutions is inherent in the three-anchor ToA-based positioning problem. The choice of the correct solution is determined from other available information, i.e., it cannot be resolved from algebra. The proposed DM reduces the three-anchor ToA-based positioning problem to the solution of a quadratic equation, i.e., Equation (12), exploiting the knowledge about the workspace of the user tag to reject the incorrect solution, and the solution of two simple equations, i.e., Equations (10) and (11).

**Figure 1:**
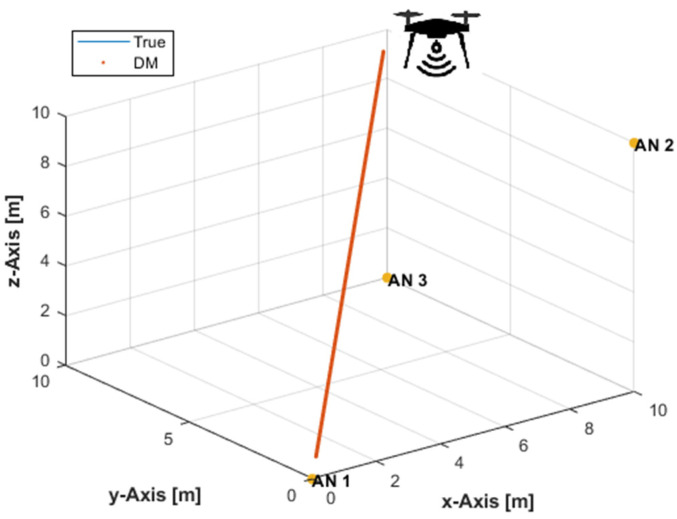
The direct method (DM) solution with three non-coplanar anchor nodes (ANs) and noise-free distance measurements.

To verify the developed DM solution, three ANs were placed at (0,0,0), (10,0,10), and (10,10,0) m, while a user tag, i.e., UAV, has moved along a linear path from position (9.5,9.5,9.5) m to position (0.5,0.5,0.5) m collecting three noise-free distance measurements from three ANs at k=361 time instants, Figure 1. In Equation (12), a solution is rejected if $x_{k}>10$ m or $x_{k}<0$ m, i.e., outside the workspace, and accepted if $0\leq x_{k}\leq 10$ m. When both solutions are accepted, their mean value is considered to be the solution for $x_{k}$, which is a non-exact solution, if no other information is available to correctly resolve the ambiguity. Figure 1 plots the DM solution superimposed on the true path. It can be seen from Figure 1 that the plot of the developed DM solution completely hides the true path plot, i.e., the developed DM solution can exactly determine the 3D position of the user tag with a non-coplanar arrangement of three ANs.

The implemented DM with three non-coplanar ANs is listed in Algorithm 1. The true distances, $r_{k,i}$, are replaced by the distance measurements, $d_{k,i}$. Thus, the results of Equations (10)–(12) are estimates of the user tag 3D position components as indicted using the hat notation. After obtaining the distance measurements, $d_{k,i}$, in step 1, two estimates for ${\hat{x}}_{k}$ are computed by Equation (12) in step 2. The solution of ${\hat{x}}_{k}$ is decided in sub-steps 2.1 or 2.2. Sub-step 2.3 applies a low-pass filter (LPF) to further reduce the effect of measurement noise, where the factor α is set to 0.7. The estimates of ${\hat{y}}_{k}$ and ${\hat{z}}_{k}$ are computed in steps 3 and 4 by Equations (10) and (11), respectively, and smoothed by an LPF. Step 5 outputs the final 3D position estimate of the user tag. If Algorithm 1 is used to verify the developed DM, the true ranges, $r_{k,i}$, are used instead of the distance measurements, $d_{k,i}$, and, therefore, the employment of the LPF would not be necessary.
**Algorithm 1.** Direct method (DM) algorithm with three non-coplanar anchor nodes (ANs)1. At time instant $t_{k}$, obtain the distance measurements, $d_{k,i}$, i∈{1,2, 3};2. Compute two estimates for ${\hat{x}}_{k}$ by Equation (12); 2.1. Reject the estimate that is placed outside the workspace, and consider the other estimate as a solution for ${\hat{x}}_{k}$; 2.2. If both estimates lay within the workspace, consider their mean value as a solution for ${\hat{x}}_{k}$ if k=1. If k>1, consider the estimate closer to ${\hat{x}}_{k-1}$ as a solution for ${\hat{x}}_{k}$; 2.3. If k>1, apply a low-pass filter (LPF) to ${\hat{x}}_{k}$ to further reduce the effect of measurement noise, i.e., ${\hat{x}}_{k}^{LPF}=\alpha \cdot {\hat{x}}_{k-1}+\left(1-\alpha \right)\cdot {\hat{x}}_{k}$;3. Compute the estimate of ${\hat{y}}_{k}$ by Equation (10). If k>1, ${\hat{y}}_{k}^{LPF}=\alpha \cdot {\hat{y}}_{k-1}+\left(1-\alpha \right)\cdot {\hat{y}}_{k}$;4. Compute the estimate of ${\hat{z}}_{k}$ by Equation (11). If k>1, ${\hat{z}}_{k}^{LPF}=\alpha \cdot {\hat{z}}_{k-1}+\left(1-\alpha \right)\cdot {\hat{z}}_{k}$;5. Output the final 3D position estimate of the user tag $x_{k}={\left[{\hat{x}}_{k},{\hat{y}}_{k},{\hat{z}}_{k}\right]}^{T}$ if k=1. If k>1, $x_{k}={\left[{\hat{x}}_{k}^{LPF},{\hat{y}}_{k}^{LPF},{\hat{z}}_{k}^{LPF}\right]}^{T}$.

With a coplanar arrangement of ANs, the ambiguity in Equation (12) becomes very difficult if not impossible to resolve without using extra information, especially at low signal-to-noise ratio (SNR) levels and measurement update rates. Therefore, an alternative solution approach is developed for this case in Section 3.2.

### 3.2. DM Solution with Three Horizontally Coplanar ANs

The horizontally coplanar arrangement of ANs can be exploited by solving first for $z_{k}$, because one of the resulting two solutions will always be negative, i.e., outside the workspace, and, therefore, can always be correctly rejected. With the same previously applied procedure, Section 3.1, $y_{k}$ can be canceled out by solving Equations (6) and (7) for $y_{k}$ to obtain an explicit expression for $x_{k}$ in terms of $z_{k}$ as

(13) $$x_{k}=\alpha_{3}+\beta_{3}\cdot z_{k},$$

where $\alpha_{3}=\frac{1}{2}\left(y_{21}\cdot A_{2}-y_{31}\cdot A_{1}\right)/\left(x_{31}\cdot y_{21}-x_{21}\cdot y_{31}\right)$ and $\beta_{3}=\left(y_{31}\cdot z_{21}-y_{21}\cdot z_{31}\right)/\left(x_{31}\cdot y_{21}-x_{21}\cdot y_{31}\right)$.

Similarly, $x_{k}$ can be canceled out by solving Equations (6) and (7) for $x_{k}$ to obtain an explicit expression for $y_{k}$ in terms of $z_{k}$ as

(14) $$y_{k}=\alpha_{4}+\beta_{4}\cdot z_{k},$$

where $\alpha_{4}=\frac{1}{2}\left(x_{31}\cdot A_{1}-x_{21}\cdot A_{2}\right)/\left(x_{31}\cdot y_{21}-x_{21}\cdot y_{31}\right)$ and $\beta_{4}=\left(x_{21}\cdot z_{31}-x_{31}\cdot z_{21}\right)/\left(x_{31}\cdot y_{21}-x_{21}\cdot y_{31}\right)$.

Substituting Equations (13) and (14) in Equation (3), we obtain two solutions for $z_{k}$ as

(15) $$z_{k}=-\frac{b_{2}\pm \sqrt{b_{2}^{2}-4a_{2}c_{2}}}{2a_{2}},$$

where $a_{2}=\beta_{3}^{2}+\beta_{4}^{2}+1$, $b_{2}=2\beta_{3}\left(\alpha_{3}-x_{1}\right)+2\beta_{4}\left(\alpha_{4}-y_{1}\right)-2z_{1}$, and $c_{2}={\left(\alpha_{3}-x_{1}\right)}^{2}+{\left(\alpha_{4}-y_{1}\right)}^{2}+z_{1}^{2}-r_{k,1}^{2}$. When solving Equation (15) with horizontally coplanar ANs, one of the solutions is always negative, i.e., outside workspace, and, thus, can always be rejected to correctly resolve the vertical position ambiguity of the user tag. After solving for $z_{k}$ by Equation (15), the DM 3D solution is completed by solving Equations (13) and (14).

**Figure 2:**
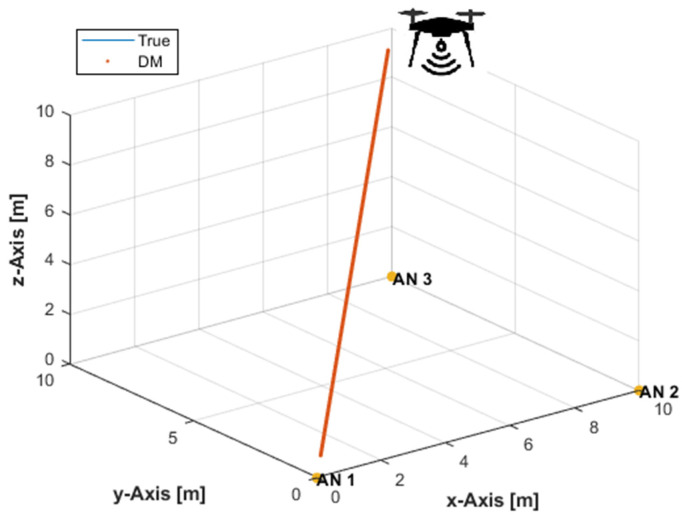
The DM solution with three coplanar ANs and noise-free distance measurements.

The same experimental settings as in Section 3.1 have been used to verify the developed DM solution with a horizontally coplanar arrangement of ANs placed at (0,0,0), (10,0,0), and (10,10,0) m. It can be seen from Figure 2 that the plot of the DM solution, superimposed on the true path, completely hides the true path plot. Therefore, the developed alternative DM solution can exactly determine the 3D position of the user tag with three coplanar ANs. Algorithm 2 lists the implemented DM with three coplanar ANs.
**Algorithm 2.** DM algorithm with three coplanar ANs1. At time instant $t_{k}$, obtain the distance measurements, $d_{k,i}$, i∈{1,2, 3};2. Compute two estimates for ${\hat{z}}_{k}$ by Equation (15); 2.1. Reject the estimate that is placed outside the workspace, and consider the other estimate as a solution for ${\hat{z}}_{k}$; 2.2. If both estimates lay within the workspace, consider their mean value as a solution for ${\hat{z}}_{k}$ if k=1. If k>1, consider the estimate closer to ${\hat{z}}_{k-1}$ as a solution for ${\hat{z}}_{k}$; 2.3. If k>1, apply an LPF to ${\hat{z}}_{k}$ to further reduce the effect of measurement noise, i.e., ${\hat{z}}_{k}^{LPF}=\alpha \cdot {\hat{z}}_{k-1}+\left(1-\alpha \right)\cdot {\hat{z}}_{k}$;3. Compute the estimate of ${\hat{x}}_{k}$ by Equation (13). If k>1, ${\hat{x}}_{k}^{LPF}=\alpha \cdot {\hat{x}}_{k-1}+\left(1-\alpha \right)\cdot {\hat{x}}_{k}$;4. Compute the estimate of ${\hat{y}}_{k}$ by Equation (14). If k>1, ${\hat{y}}_{k}^{LPF}=\alpha \cdot {\hat{y}}_{k-1}+\left(1-\alpha \right)\cdot {\hat{y}}_{k}$;5. Output the final 3D position estimate of the user tag $x_{k}={\left[{\hat{x}}_{k},{\hat{y}}_{k},{\hat{z}}_{k}\right]}^{T}$ if k=1. If k>1, $x_{k}={\left[{\hat{x}}_{k}^{LPF},{\hat{y}}_{k}^{LPF},{\hat{z}}_{k}^{LPF}\right]}^{T}$.

## 4. Particle Filtering for ToA-Based 3D Positioning

The mathematical foundations of the PF for positioning were presented in [111]. Therefore, only the PF implementation will be discussed here. The PF [112] solves the ToA-based positioning problem using random samples called particles, i.e., user tag 3D position candidates, instead of parametric distributions to overcome the limitations caused by the Gaussian assumption. In this way, the PF can simultaneously deal with nonlinear models and non-Gaussian/multimodal distributions. The PF approximates the posterior PDF, $p(x_{k}|d_{k,i})$, as a weighted combination of particles [111].

(16) $$p(x_{k}|d_{k,i})\approx \sum_{p=1}^{P}w_{k}^{p}\delta \left(x_{k}-x_{k}^{p}\right),\;i\in \left\{1,2,\;3\right\},$$

where $x_{k}$ is the state, i.e., the unknown user tag 3D position as previously defined, δ(x) is the Dirac delta function, $w_{k}^{p}$ is the weight of the particle, $x_{k}^{p}$, P is the total number of particles, and the superscript p=1,2,…,P denotes the particle number. All weights sum up to unity to represent a valid posterior PDF. Generally, the more particles are used (to a certain limit), the faster the PF will converge to the true position.

Algorithm 3 lists the implemented PF. In the beginning, no information is available about the user tag 3D position. Therefore, some P particles are generated by the PF and uniformly distributed in the whole workspace. When three distance measurements, $d_{k,i}$, from three ANs are available at the first time instant, $t_{k}$, k=1, the weight, $w_{k}^{p}$, of each particle, $x_{k}^{p}$, is then computed [111].

(17) $$w_{k}^{p}=1/\sum_{i}{\left(d_{k,i}-d_{k,i}^{p}\right)}^{2},\;i\in \left\{1,2,\;3\right\},$$

where $d_{k,i}^{p}$ is the *i*-th distance or range, at the time instant, $t_{k}$, from the particle $x_{k}^{p}$ to AN *i*, i∈{1,2, 3}. Thus, $d_{k,i}^{p}=∥a_{i}-x_{k}^{p}∥$, i∈{1,2, 3}, represents the measurement model. The weight of each particle is inversely proportional to the similarity metric used, which is the sum of squared distances between $d_{k,i}$ and $d_{k,i}^{p}$.

A number L<P, of the best-weighted particles, is selected and their weights are normalized to determine the state, $x_{k}$, as the weighted trimmed average estimate (WTAE) [111]

(18) $$x_{k}=\sum_{p=1}^{L}x_{k}^{p}w_{k}^{p}/\sum_{p=1}^{L}w_{k}^{p}.$$

The estimation of the user tag 3D position, $x_{k}$, at the first time instant, $t_{k}$, k=1, completes the initialization phase of the PF. The PF algorithm continues by repeating steps 1–3, at every time instant, $t_{k}$, k>1.

In the first step, prediction, the PF generates particles in new locations to account for the dynamics of the user tag. These particles are uniformly distributed and are generated according to the resampling procedure [111]

(19) $$x_{k}^{p}\sim U\left(x_{k-1}-R,\;x_{k-1}+R\right),\;k>1,$$

where $R=\left[\begin{array}{ccc} R_{x} & R_{y} & R_{z} \end{array}\right]$ is the resampling space, and $R_{x}$, $R_{y}$, and $R_{z}$, are the resampling ranges in the *x*-, *y*-, and *z*-directions, respectively, and assumed to be equal, i.e., $R_{x}=R_{y}=R_{z}=R$.

In other words, the prediction step generates new particles from a proposal distribution approximating the posterior PDF, in which the new particles are uniformly distributed within a cuboid or bounding box with a side length of 2R and center at the previous state estimate $x_{k-1}$. The value of R is determined so that to consider the maximum expected displacement in the *x*-, *y*-, and *z*-directions during the measurement sampling time. The value of R should also account for extreme situations such as sudden or unexpected movements to enable recovery from incorrect predictions. The cloud of particles is, thus, continuously updated to simultaneously guarantee a good representation of the posterior PDF and an acceptable estimation accuracy while avoiding degeneracy of the PF over time. When new distance measurements, $d_{k,i}$, are available, the weights of the new particles are calculated by Equation (17) and then a new state estimate is computed by Equation (18). The implemented PF used a constant number P=1000 particles. The PF estimated the state, $x_{k}$, by Equation (18) using the 10% best-weighted particles, i.e., L=0.1·P.

**Figure 3:**
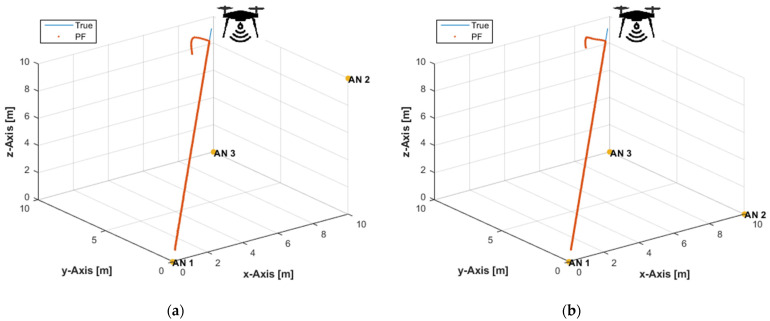
The PF solution with noise-free distance measurements and: (**a**) Three non-coplanar ANs; (**b**) Three coplanar ANs.

The developed PF has been verified with the same experimental settings as in Section 3.1 and Section 3.2 using non-coplanar ANs and coplanar ANs, Figure 3a and Figure 3b respectively. It can be seen from Figure 3 that the PF converges quickly to the true path after a few time instants. The successful operation of the PF with any AN arrangement is due to its ability to find the correct position candidates if the cloud of particles is correctly generated in the state space of interest. It is assumed that the PF will always generate some particles near the true user tag position and, thus, can overcome ambiguities and poor geometries.
**Algorithm 3.** Particle filter (PF) algorithm for ToA-based 3D positioning with three ANs**0. Initialization:** Generate some P particles uniformly distributed in the whole workspace. Compute the weight of each particle $x_{k}^{p}$ according to Equation (17), when distance measurements, $d_{k,i}$, are available at the time instant $t_{k}$, k=1. Estimate the 3D position of the user tag, $x_{k}$, according to Equation (18).**1. Prediction:** Generate new particles according to Equation (19).**2. Update:** Compute the weight, $w_{k}^{p}$, of each particle, $x_{k}^{p}$, according to Equation (17), when distance measurements, $d_{k,i}$, are available at the time instant $t_{k}$, k>1.**3. State Estimation:** Estimate the user tag 3D position, $x_{k}$, according to Equation (185).Set k=k+1 and repeat from step 1.

The horizontally coplanar ANs are ill-posed to perform 3D positioning using matrix-based approaches such as LS and ML algorithms, because the rank of the data matrix is reduced and, thus, the vertical position, ${\hat{z}}_{k}$, cannot be reliably estimated. Therefore, LS and ML algorithms require non-coplanar arrangements to prevent this situation, and at least four ANs to simplify/linearize the system of nonlinear (quadratic) equations to remove the square root, whereas the PF can keep a cloud of particles with similar horizontal positions and different vertical positions. As time evolves the mechanism of the PF enables convergence to the correct vertical position. In other words, the PF can scan the correct 3D space to overcome the low observability of the vertical position.

## 5. Simulation Results

The performance of the proposed algorithms was evaluated by computer simulations using MATLAB. The root mean square error (RMSE) was used as the performance metric. The performance was investigated upon varying SNR levels (30, 35, and 40 dB) and measurement update rates (4, 8, and 16 Hz). A ranging-based UWB localization system with a high frequency of 20 Hz was used in [24]. A four-anchor UWB system ranging up to 125 m at a measurement update rate of 40 Hz was deployed in [113]. Reference [114] reported a UWB TWR at 80 Hz. UWB TWR schemes were designed in [56] and reached a minimum and maximum practical measurement update rate of 62 and 372 Hz, respectively, where reliable ranging up to 80 m and occasionally up to 220 m was obtained. The distance measurements, $d_{k,i}$, were simulated as true value plus a zero-mean Gaussian noise proportional to the true distance. Therefore, an LPF was applied to the measurements to reduce noise and remove outliers. The SNR at time instant, $t_{k}$, of the distance measurement, $d_{k,i}$, and the variance of the Gaussian noise, $\sigma_{k,i}^{2}$, are, respectively, defined [111].

(20) $$SNR=10log\frac{{\left(∥a_{i}-x_{k}∥\right)}^{2}}{\sigma_{k,i}^{2}}\;[\mathrm{dB}],$$

(21) $$\sigma_{k,i}^{2}=\frac{{\left(∥a_{i}-x_{k}∥\right)}^{2}}{10^{SNR/10}}\;[m^{2}].$$

Two kinds of navigation scenarios were investigated [115]: (1) the target pursuit scenario [116,117,118], e.g., autonomous docking, in which the UAV is required to navigate to a predetermined position; (2) the circumnavigation scenario [119,120], in which the UAV circles around a stationary (or moving) target position. In the target pursuit scenario, two paths were considered: (1) a 3D linear path, Section 5.1; (2) a horizontal linear path, Section 5.2. In the circumnavigation scenario, a horizontal circular path was investigated, Section 5.3. Non-coplanar and coplanar arrangements of ANs were considered. Identical position components of the ANs’ locations were avoided by applying differences of 10 cm when necessary to resemble realistic conditions. All RMSE results were obtained over 100 ensemble runs for all user tag positions.

### 5.1. Three-Dimensional Linear Path

**Figure 4:**
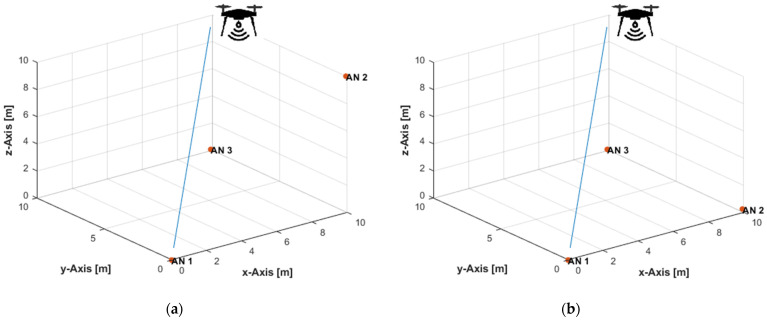
Three-dimensional (3D) linear path with: (**a**) Non-coplanar ANs; (**b**) Coplanar ANs.

Three ANs were placed at (0,0,0), (10,0.1,10), and (9.9,10,0.1) m, in the non-coplanar configuration (Figure 4a). In the coplanar arrangement, the ANs were placed at (0,0,0), (10,0.1,0.2), and (9.9,10,0.1) m (Figure 4b). The UAV, equipped with a user tag, moved over a linear path from position (9.5,9.5,9.5) m to position (0.5,0.5,0.5) m (Figure 4) at constant velocities of 0.1 m/s in the *x*-, *y*-, and *z*-directions. Table 1 lists the total number of ToA measurements obtained from each AN along the path, and the traveled distances in the *x*-, *y*-, and *z*-directions between any two successive measurement times, at the measurement update rates of 4, 8, and 16 Hz. The maximum true range along the path was 16.45 m. Thus, the corresponding maximum standard deviations of the distance measurement noise, according to Equation (21), at the investigated SNR levels of 30, 35, and 40 dB were, respectively, 52, 29, and 16 cm. In [71], up to 10 cm UWB ranging error was reported for a maximum range of up to 50 m. A range measurement accuracy of about 10 cm was achieved over a working range of a few hundred meters in [114], where the ranging accuracy had the same level irrespective of the tag–anchor distance.

**Figure 5:**
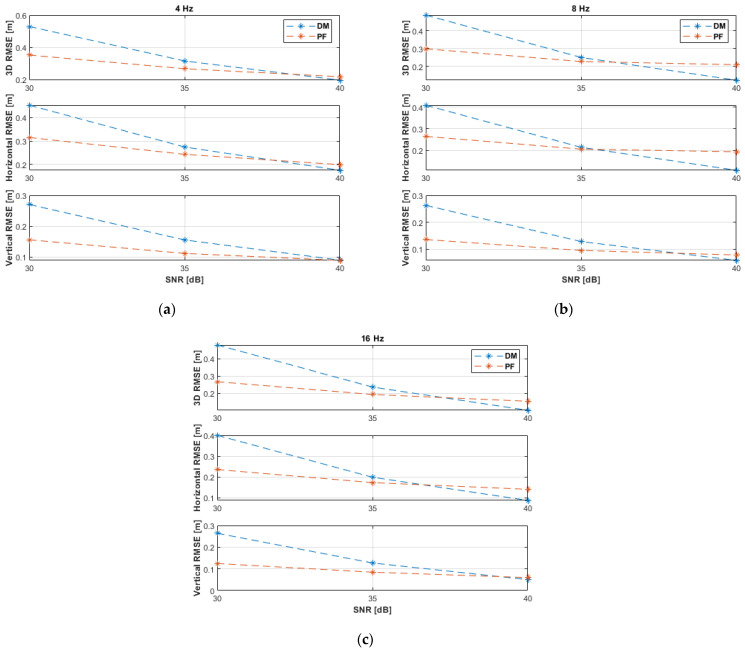
Root mean square error (RMSE) results of the 3D linear path experiments with non-coplanar anchor nodes (ANs) at measurement update rates of: (**a**) 4 Hz; (**b**) 8 Hz; (**c**) 16 Hz.

Figure 5 shows the 3D, horizontal, and vertical RMSE of the DM and PF solutions with non-coplanar ANs at the investigated SNR levels and measurement update rates. The PF results at 4 Hz were obtained with a resampling range, R, of 20 cm, while results at 8 and 16 Hz were obtained with R=10 cm. All results, rounded to two significant digits, are also summarized in Table 2. As expected, the performance improves, in general, with increasing SNR levels and measurement update rates. Increasing the SNR level is more significant to the DM algorithm than to the PF solution, i.e., the DM method is more sensitive to measurement noise, while the PF shows a more robust behavior. At the highest investigated SNR level of 40 dB, the DM outperformed the PF. At 40 dB and 16 Hz, the DM achieved a horizontal accuracy of 9 cm and a vertical accuracy of 5 cm, while the PF achieved 14 cm and 6 cm, respectively. The PF outperformed the DM at the SNR levels of 30 and 35 dB.

**Figure 6:**
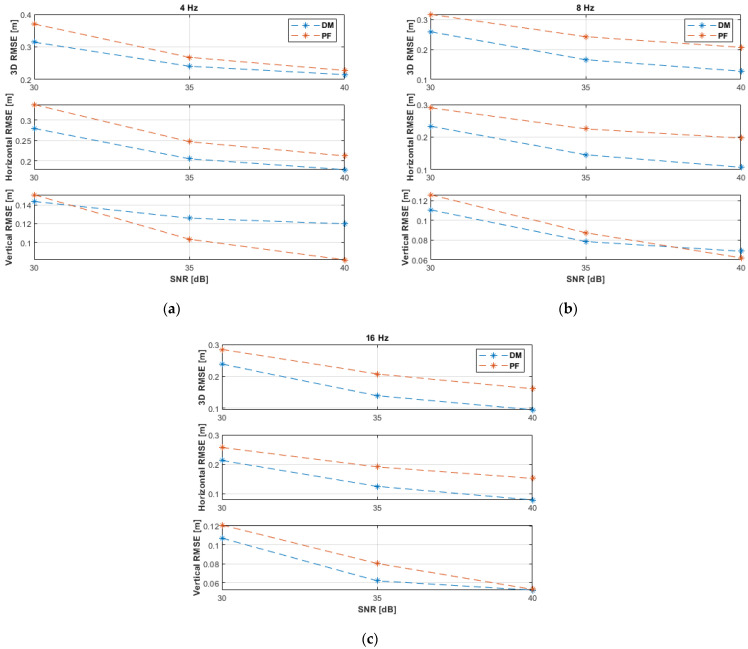
RMSE results of the 3D linear path experiments with coplanar ANs at measurement update rates of: (**a**) 4 Hz; (**b**) 8 Hz; (**c**) 16 Hz.

The experiments have been repeated using the same settings with coplanar ANs. The results are depicted in Figure 6 and summarized in Table 3. The DM (Algorithm 2) outperformed the PF in the horizontal plane and the overall accuracy, i.e., 3D space, at all investigated SNR levels and measurement update rates. Generally, the DM achieved better vertical accuracy at measurement update rates of 8 and 16 Hz. At 40 dB and 16 Hz, the DM achieved a horizontal accuracy of 8 cm and a vertical accuracy of 5 cm, while the PF achieved 15 cm and 5 cm, respectively, i.e., similar to the results with non-coplanar ANs. The comparison of results in Table 2 and Table 3 reveals that the PF achieved similar results irrespective of the arrangement of ANs, i.e., the PF is robust against varying conditions, while the performance of the DM improved remarkably with coplanar ANs at the SNR levels of 30 and 35 dB, i.e., the DM is sensitive to varying conditions. The results attained by the DM with coplanar ANs (Figure 6 and Table 3) were better than with non-coplanar ANs (Figure 5 and Table 2) because in the former case the UAV was moving closer towards the ANs, i.e., causing less measurement noise, while in the latter case the UAV was moving away from the second AN.

### 5.2. Horizontal Linear Path

**Figure 7:**
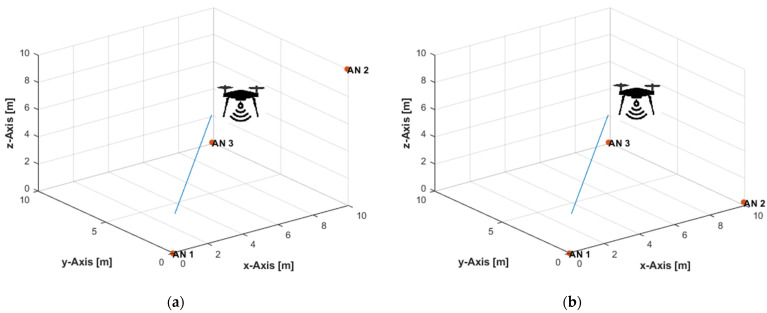
Horizontal linear path with: (**a**) Non-coplanar ANs; (**b**) Coplanar ANs.

The UAV moved over a horizontal linear path from position (9.5,9.5,2.5) m to position (0.5,0.5,2.5) m (Figure 7) at a constant height of 2.5 m and constant velocities of 0.1 m/s in the *x*- and *y*-directions. The total number of measurements and traveled distances, in the *x*- and *y*-directions only, over the path at the investigated measurement update rates were identical to that listed in Table 1. The maximum true range over the path was 13.67 m. Thus, the corresponding maximum standard deviations of the distance measurement errors, according to Equation (21), at the investigated SNR levels of 30, 35, and 40 dB were, respectively, 43, 24, and 14 cm. Due to the constant height motion, the overall UAV dynamics in the experiments are less than those in the 3D linear path experiments presented in Section 5.1. All PF results were obtained with a resampling range, R, of 10 cm.

**Figure 8:**
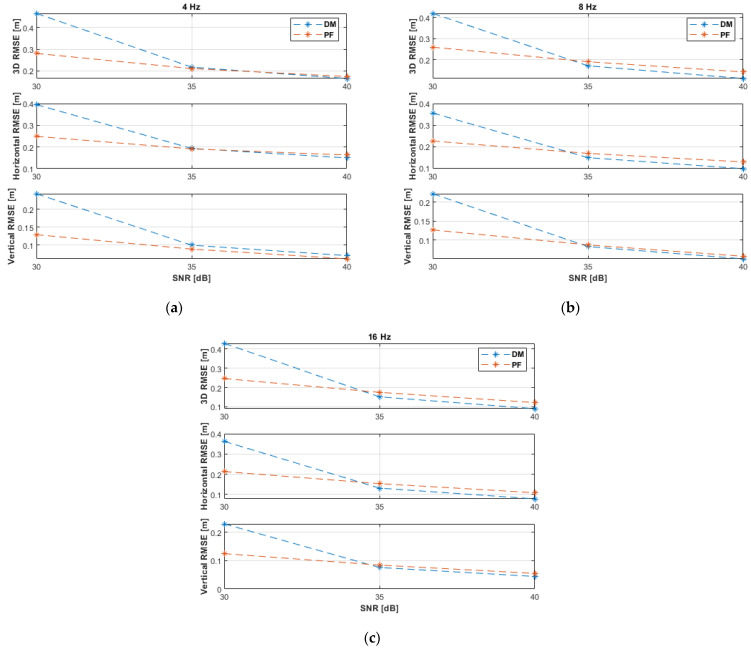
RMSE results of the horizontal linear path experiments with non-coplanar ANs at measurement update rates of: (**a**) 4 Hz; (**b**) 8 Hz; (**c**) 16 Hz.

The RMSE results of the experiments with non-coplanar ANs are shown in Figure 8 and listed in Table 4. It can be noticed that the result trends are similar to those of the 3D linear path experiments (Figure 5 and Table 2). However, the performance of the DM and PF in the horizontal linear path experiments, as can be seen from Figure 8 and Table 4, are slightly better due to the overall lower UAV dynamics, since no motion occurs in the vertical direction, and, therefore, position estimation is accomplished with fewer uncertainties. Moreover, the DM can already outperform the PF earlier than in the 3D linear path experiments, i.e., starting from an SNR level of 35 dB and a measurement update rate of 8 Hz. At 40 dB and 16 Hz, the DM achieved a horizontal accuracy of 8 cm and a vertical accuracy of 4 cm, while the PF achieved 11 cm and 6 cm, respectively.

**Figure 9:**
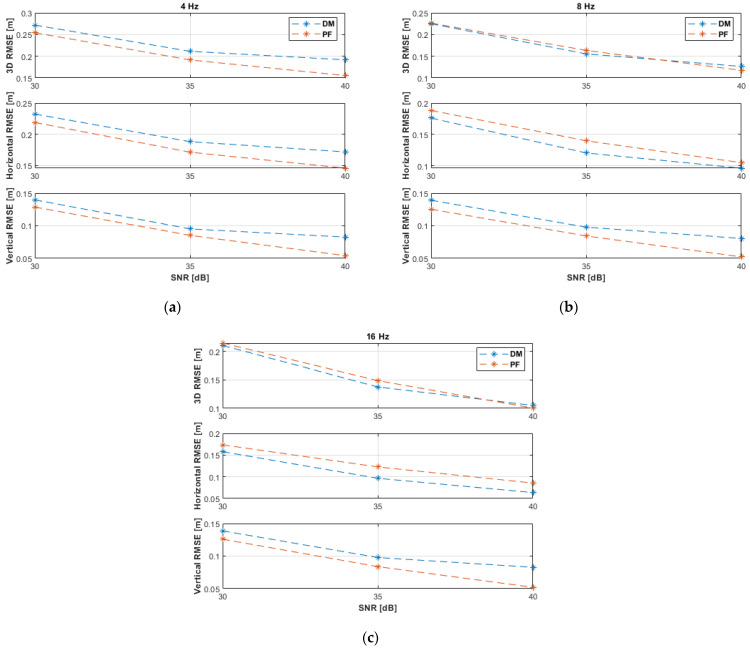
RMSE results of the horizontal linear path experiments with coplanar ANs at measurement update rates of: (**a**) 4 Hz; (**b**) 8 Hz; (**c**) 16 Hz.

The experimental results with coplanar ANs are illustrated in Figure 9 and recorded in Table 5. The performance with coplanar ANs is somewhat better than with non-coplanar ANs (Figure 8 and Table 4) because in the former case the UAV path was closer to the (coplanar) ANs. Therefore, the performance of the DM is, again, remarkably improved at the SNR level of 30 dB. Similar accuracies were attained by both the DM and PF solutions with coplanar ANs. The PF achieved, in general, 1–3 cm better accuracies than the DM. The horizontal positioning accuracy of the DM was 1–3 cm better at 8 and 16 Hz. At 40 dB and 16 Hz, the DM achieved a horizontal accuracy of 6 cm and a vertical accuracy of 8 cm, while the PF achieved 9 cm and 5 cm, respectively.

### 5.3. Horizontal Circular Path

**Figure 10:**
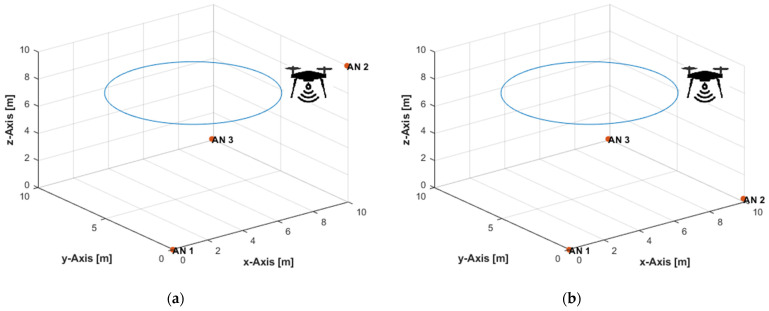
Horizontal circular path with: (**a**) Non-coplanar ANs; (**b**) Coplanar ANs.

The UAV moved over a horizontal circular path, with a radius of 4 m and center at (5,5,7.5) m (Figure 10), at a constant height of 7.5 m and a constant angular velocity of $\frac{\pi }{50}$ rad/s. The UAV completed a single full round starting from position (9,5,7.5) m. The corresponding velocities in the *x*- and *y*-directions varied between a minimum of 0.008 m/s and a maximum of 0.25 m/s. The total number of ToA measurements obtained over the path at the measurement update rates of 4, 8, and 16 Hz were, respectively, 401, 801, and 1601. The maximum true range over the path was 13.37 m. Thus, the corresponding maximum standard deviations of the measurement errors, according to Equation (21), at the investigated SNR levels of 30, 35, and 40 dB were, respectively, 42, 24, and 13 cm. The PF results at 4 Hz were obtained with a resampling range, R, of 20 cm, while results at 8 and 16 Hz were obtained with R=10 cm.

**Figure 11:**
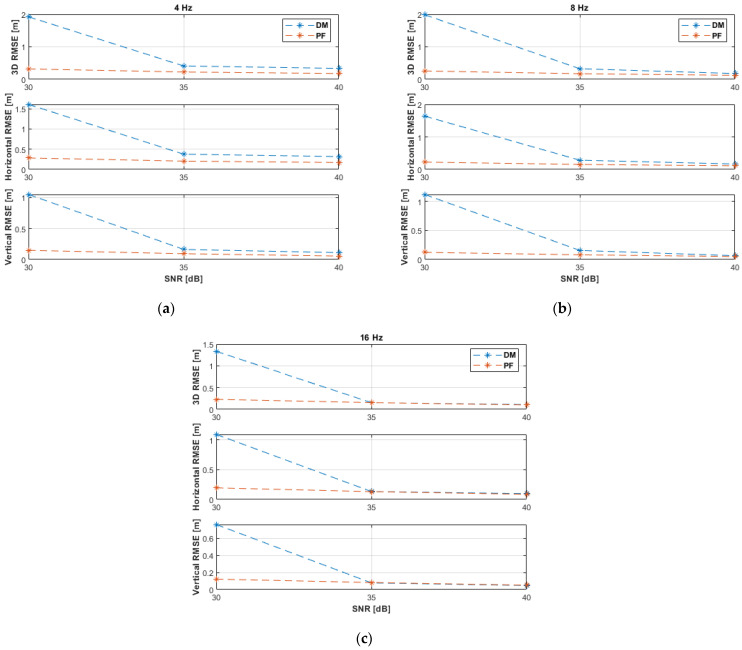
RMSE results of the horizontal circular path experiments with non-coplanar ANs at measurement update rates of: (**a**) 4 Hz; (**b**) 8 Hz; (**c**) 16 Hz.

The UAV experienced higher dynamics during the circular motion due to the varying velocities, in magnitude and direction, and because the maximum velocity was higher than in the previous two experimental paths. High motion dynamics and low SNR levels increased the positioning uncertainty. The PF can minimize the impact of these factors during the prediction step (at low measurement update rates) by increasing the size of the resampling range, R. The simulation results with non-coplanar ANs (Figure 11 and Table 6) show that the DM was unable to reliably estimate the UAV position at the SNR level of 30 dB due to higher uncertainties. The DM improved its performance significantly at higher SNR levels, i.e., from 35 dB. The PF outperformed the DM at the measurement update rates of 4 and 8 Hz. Both algorithms attain (almost) identical accuracies at 16 Hz and from 35 dB. At 40 dB and 16 Hz, the DM achieved a horizontal accuracy of 10 cm and a vertical accuracy of 5 cm, while the PF achieved 9 cm and 5 cm, respectively.

**Figure 12:**
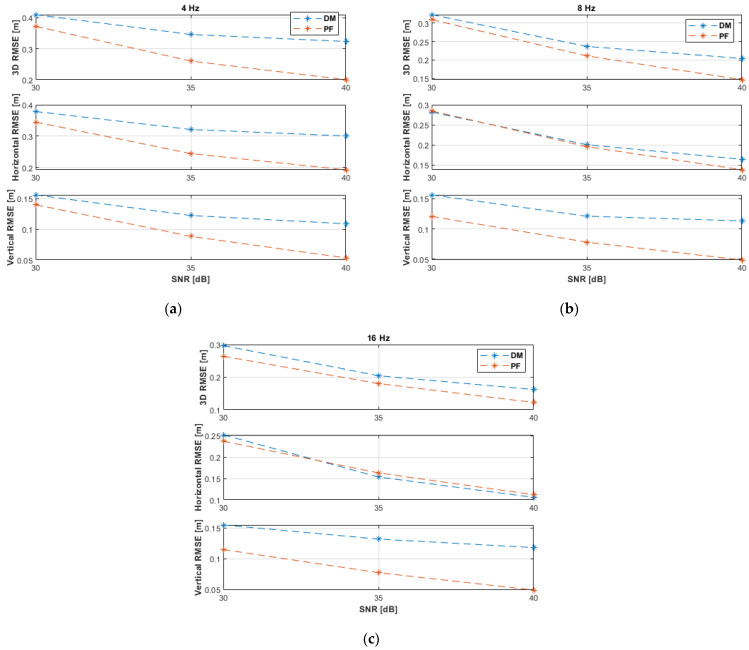
RMSE results of the horizontal circular path experiments with coplanar ANs at measurement update rates of: (**a**) 4 Hz; (**b**) 8 Hz; (**c**) 16 Hz.

The results with coplanar ANs appear in Figure 12 and Table 7. The PF almost outperformed the DM. Compared to the non-coplanar case (Figure 11 and Table 6), the performance of the DM was improved with the coplanar ANs at the SNR level of 30 dB and all investigated measurement update rates, 35 dB and update rates of 4 and 8 Hz, and 40 dB and 4 Hz. This is because, with higher measurement noise, the DM will be initially less capable to correctly resolving the ambiguity of the *x*-coordinate (both *x* solutions might be acceptable, and their mean value would be considered) of the UAV 3D position, Equation (12), with the non-coplanar ANs. With coplanar ANs the horizontal plane is the plane of symmetry of the ANs’ layout, which is a more effective plane of symmetry to resolve the ambiguity of the *z*-coordinate of the UAV 3D position, Equation (15), since there will always be a negative *z* solution to be rejected and, thus, the other correct *z* solution will always be considered without averaging, irrespective of the SNR level.

All PF results and the DM results (at better measurement conditions, i.e., 35 dB at 16 Hz, and 40 dB at 8 and 16 Hz) were a bit less accurate than the non-coplanar experimental results (Figure 11 and Table 6) because the second AN is more distant to the circular path in the coplanar case than in the non-coplanar case.

### 5.4. Remarks

The discussions, investigations, and simulation assumptions in this study are directly applicable to performance evaluation/prediction and system engineering contexts. Bandwidth limitations, of the network channel connecting ANs and user tag, can cause random delays of the received measurements that may impair the applicability of the proposed DM and PF solutions [121]. Therefore, these delays must be accounted for in fielded positioning systems.

The mean computation time required for a single run of the DM, $T_{DM}$, and the PF with 1000 particles, $T_{PF}$, was estimated on an Intel Core i7-8565U CPU at 1.8 GHz with 24 GB RAM running MATLAB R2020a. The estimated $T_{DM}$ and $T_{PF}$ values on the platform were less than 0.13 and 5 ms, i.e., allowing the DM and PF to run at more than 7.6 and 0.2 kHz, respectively, within the MATLAB environment. The developed DM would be a potential positioning solution, due to its high performance and low computational complexity, for high-frequency positioning systems with high-accuracy ranging capacity that may appear in the future. The developed PF is a perfect parallel computing case and is suitable for implementation on platforms, such as the compute unified device architecture (CUDA).

The DM is generally more sensitive than the PF to varying conditions such as SNR levels, measurement update rates, arrangement of ANs, and target motion dynamics. The PF is more robust against such variations. With high levels of SNR and measurement update rate associated with low levels of motion dynamics, i.e., with high certainty measurement conditions, the DM outperforms the PF, because the DM is an exact (deterministic) solution. The advantage of the PF occurs at higher uncertainties, e.g., lower levels of SNR and measurement update rate, and higher motion dynamics, due to its probabilistic mechanism. With higher uncertainties, the PF accommodates the situation by increasing the size of the resampling range, R. Hence its robust performance over varying measurement conditions.

The control of UAVs during autonomous flights can be achieved in the short term to some extent by onboard inertial sensors, which provide information about variables such as position, velocity, acceleration, and attitude. In the long term, an accurate estimation of these variables is not feasible due to the drift of inertial sensors, which causes time-discrete integration errors. Therefore, an absolute positioning reference is required to ensure that the take-off, hovering, trimming, and landing of UAVs are completely automatic. During autonomous landing, the UAV will descend when its horizontal position is within the proximity of the center of the landing pad, i.e., horizontal threshold. The throttle will cut off automatically when the vertical position is below a certain vertical threshold, e.g., 20 cm or less, which is adequate for autonomous docking [34]. UAVs are usually uncontrollable at heights less than 20 cm due to the near-ground effect, caused by the wind effect of the UAV’s propellers on the ground [122,123].

## 6. Conclusions

Simulation results corroborate the viability of the developed DM and PF algorithms to solve the three-anchor ToA-based 3D positioning problem and to overcome the poor vertical position observability in the case of the horizontally coplanar arrangement of ANs. The developed solutions require minimal assumptions, hardware costs, and computational complexity. Therefore, they are practical for, e.g., UAV applications, especially if the UAV needs to accurately land and navigate short distances in GNSS-denied spaces or when other more precise, expensive, and sophisticated positioning techniques could not be used. The proposed solutions are also important for increasing the availability of positioning services with acceptable accuracy levels if additional, i.e., more than three, ToA measurements are not available due to, e.g., AN failure or unreliable measurements. The DM is especially a useful low-complexity solution to be adopted in radar networks, to solve the positioning problem as well as to prune invalid hypotheses in advance, since the number of hypotheses becomes significantly high for an increasing number of targets and/or receiving stations [124].

The real-time availability of measurements and consistency of noise distribution are crucial conditions to enable the proper working of the developed DM and PF algorithms. These conditions may be violated in practice, resulting in degradation of estimation performance or failure of the algorithms, unless proper tuning measures are implemented, leading to modifications to the proposed algorithms to accommodate these situations.

Simulations always include assumptions and simplifications, which are necessary for the proof of concepts but may not perfectly resemble the real world. Therefore, there is still work remaining to test the developed algorithms with real-world experiments to account for, e.g., signal dampening, interference, and reflections. Real-world experimentation may present new and unforeseen problems or may require modification of some assumptions.

Although UAV flight is simpler indoors [125] due to the absence of, e.g., wind, fog, and rain, accurate and reliable wireless positioning indoors is still a major challenge in the presence of multipath and non-line of sight (NLoS) signal effects. Therefore, further research efforts are needed to overcome these effects.

The DM and PF solutions can be extended to work for cooperative positioning, where a user tag with an estimated position can be employed as AN and, thus, more measurements and better geometries can be provided for positioning other user tags. Exploring and testing an UWB-based formation flight is an interesting extension of the present work. Further interesting research topics include autonomous docking on a moving platform, circumnavigation around a moving target position, and use of artificial intelligence and machine learning algorithms to further improve the positioning solutions.

Future research should pay attention to the investigation and performance comparison of the PF algorithm and random search optimization algorithms, i.e., evolutionary computing approaches such as the pigeon-inspired optimization [126], to obtain a better understanding of differences and similarities.

## Figures and Tables

**Table 1:** The total number of measurements obtained over the three-dimensional (3D) linear path, and the corresponding traveled distances in the *x*-, *y*-, and *z*-directions between any two successive measurement times, at the investigated measurement update rates.

Measurement Update Rate (Hz)	Total No. of Measurements	Traveled Distance (cm)
4	361	2.5
8	721	1.25
16	1441	0.625

**Table 2:** Root mean square error (RMSE) results of the 3D linear path experiments with non-coplanar anchor nodes (ANs).

RMSE (m)	30 dB		35 dB		40 dB		Measurement Update Rate
	DM	PF	DM	PF	DM	PF	
3D	0.53	0.35	0.32	0.27	0.20	0.22	
Horizontal	0.45	0.31	0.27	0.24	0.17	0.20	4 Hz
Vertical	0.27	0.16	0.16	0.11	0.09	0.09	
3D	0.49	0.30	0.25	0.23	0.12	0.21	
Horizontal	0.41	0.26	0.21	0.21	0.11	0.19	8 Hz
Vertical	0.26	0.14	0.13	0.09	0.06	0.08	
3D	0.48	0.27	0.24	0.19	0.10	0.15	
Horizontal	0.40	0.24	0.20	0.17	0.09	0.14	16 Hz
Vertical	0.26	0.12	0.13	0.08	0.05	0.06	

**Table 3:** RMSE results of the 3D linear path experiments with coplanar ANs.

RMSE (m)	30 dB		35 dB		40 dB		Measurement Update Rate
	DM	PF	DM	PF	DM	PF	
3D	0.31	0.37	0.24	0.27	0.21	0.23	
Horizontal	0.28	0.34	0.20	0.25	0.18	0.21	4 Hz
Vertical	0.14	0.15	0.13	0.10	0.12	0.08	
3D	0.26	0.32	0.17	0.24	0.13	0.21	
Horizontal	0.23	0.29	0.15	0.23	0.11	0.20	8 Hz
Vertical	0.11	0.13	0.08	0.09	0.07	0.06	
3D	0.24	0.28	0.14	0.21	0.10	0.16	
Horizontal	0.21	0.26	0.12	0.19	0.08	0.15	16 Hz
Vertical	0.11	0.12	0.06	0.08	0.05	0.05	

**Table 4:** RMSE results of the horizontal linear path experiments with non-coplanar ANs.

RMSE (m)	30 dB		35 dB		40 dB		Measurement Update Rate
	DM	PF	DM	PF	DM	PF	
3D	0.46	0.28	0.22	0.21	0.17	0.17	
Horizontal	0.40	0.25	0.19	0.19	0.15	0.16	4 Hz
Vertical	0.24	0.13	0.10	0.09	0.07	0.06	
3D	0.42	0.26	0.17	0.19	0.11	0.14	
Horizontal	0.36	0.23	0.15	0.17	0.10	0.13	8 Hz
Vertical	0.22	0.13	0.08	0.09	0.05	0.06	
3D	0.43	0.25	0.15	0.18	0.09	0.12	
Horizontal	0.36	0.21	0.13	0.15	0.08	0.11	16 Hz
Vertical	0.23	0.13	0.08	0.08	0.04	0.06	

**Table 5:** RMSE results of the horizontal linear path experiments with coplanar ANs.

RMSE (m)	30 dB		35 dB		40 dB		Measurement Update Rate
	DM	PF	DM	PF	DM	PF	
3D	0.27	0.25	0.21	0.19	0.19	0.16	
Horizontal	0.23	0.22	0.19	0.17	0.17	0.15	4 Hz
Vertical	0.14	0.13	0.10	0.09	0.08	0.05	
3D	0.22	0.23	0.16	0.16	0.13	0.12	
Horizontal	0.18	0.19	0.12	0.14	0.10	0.10	8 Hz
Vertical	0.14	0.13	0.10	0.08	0.08	0.05	
3D	0.21	0.21	0.14	0.15	0.11	0.10	
Horizontal	0.16	0.17	0.10	0.12	0.06	0.09	16 Hz
Vertical	0.14	0.13	0.10	0.08	0.08	0.05	

**Table 6:** RMSE results of the horizontal circular path experiments with non-coplanar ANs.

RMSE (m)	30 dB		35 dB		40 dB		Measurement Update Rate
	DM	PF	DM	PF	DM	PF	
3D	1.92	0.32	0.41	0.23	0.34	0.18	
Horizontal	1.61	0.28	0.38	0.21	0.32	0.17	4 Hz
Vertical	1.05	0.15	0.16	0.09	0.11	0.06	
3D	1.99	0.26	0.33	0.17	0.18	0.12	
Horizontal	1.64	0.23	0.28	0.15	0.17	0.11	8 Hz
Vertical	1.12	0.12	0.16	0.08	0.07	0.05	
3D	1.33	0.23	0.15	0.16	0.11	0.10	
Horizontal	1.10	0.20	0.13	0.13	0.10	0.09	16 Hz
Vertical	0.76	0.12	0.08	0.08	0.05	0.05	

**Table 7:** RMSE results of the horizontal circular path experiments with coplanar ANs.

RMSE (m)	30 dB		35 dB		40 dB		Measurement Update Rate
	DM	PF	DM	PF	DM	PF	
3D	0.41	0.37	0.35	0.26	0.32	0.20	
Horizontal	0.38	0.34	0.32	0.24	0.30	0.19	4 Hz
Vertical	0.16	0.14	0.12	0.09	0.11	0.05	
3D	0.32	0.31	0.24	0.21	0.20	0.15	
Horizontal	0.28	0.28	0.20	0.20	0.16	0.14	8 Hz
Vertical	0.16	0.12	0.12	0.08	0.11	0.05	
3D	0.30	0.26	0.20	0.18	0.16	0.12	
Horizontal	0.25	0.24	0.15	0.16	0.11	0.11	16 Hz
Vertical	0.16	0.11	0.13	0.08	0.12	0.05	

## Data Availability

Not applicable.

## Abbreviations

2D	Two-dimensional
3D	Three-dimensional
5G	Fifth-generation
6G	Sixth-generation
AN	Anchor node
AoA	Angle of arrival
CF	Closed-form
CPU	Central processing unit
CRLB	Cramer–Rao lower bound
CUDA	Compute unified device architecture
DM	Direct method
DME	Distance measurement equipment
FAA	Federal Aviation Administration
FoV	Field of view
GNSS	Global navigation satellite system
GPS	Global positioning system
IoT	Internet of things
LPF	Low-pass filter
LS	Least-squares
MIMO	Multiple-input multiple-output
ML	Maximum likelihood
mmWave	Millimeter-wave
*N*-D	*N*-dimensional
NLoS	Non-line of sight
PDF	Probability density function
PF	Particle filter
RAM	Random-access memory
RFID	Radiofrequency identification
RHS	Right-hand side
RMSE	Root mean square error
RSS	Received signal strength
RTK	Real-time kinematic
RToF	Round time of flight
RTT	Round-trip time
SNR	Signal-to-noise ratio
TDoA	Time difference of arrival
THz	Terahertz
ToA	Time of Arrival
TSoA	Time sum of arrival
TWR	Two-way ranging
UAS	Unmanned aircraft systems
UAV	Unmanned aerial vehicle
UWB	Ultra-wideband
VLC	Visible light communication
VTOL	Vertical take-off and landing
WLS	Weighted least-squares
WTAE	Weighted trimmed average estimate
